# Anti-Invasion and Antimetastatic Effects of Porcine Recombinant NK-lysin on SMMC-7721 Human Hepatocellular Carcinoma Cells

**DOI:** 10.1155/2019/5318729

**Published:** 2019-04-21

**Authors:** Kuohai Fan, Wenjuan Du, Zhirui Wang, Ajab Khan, Hongquan Li, Junbing Jiang

**Affiliations:** ^1^Laboratory Animal Center, Shanxi Agricultural University, Taigu, Shanxi, China; ^2^College of Animal Science and Veterinary Medicine, Shanxi Agricultural University, Taigu, Shanxi, China; ^3^Plastic & Reconstructive Surgery, University of Colorado Denver, Denver, CO, USA

## Abstract

The high invasion and metastasizing abilities of hepatocellular carcinoma (HCC) are the primary reasons for the high mortality rate of patients. Therefore, identification of agents to inhibit invasion and metastasis is very important for treatment of HCC. We analyzed the anti-invasion and antimetastatic effects of porcine recombinant NK-lysin, which was designed and expressed* in vitro* by our research group, on SMMC-7721 hepatocellular carcinoma cells via wound-healing assays, adhesion assays, invasion assays, real-time polymerase chain reaction (PCR), and Western blot analysis. MTT assay results indicated that NK-lysin inhibited the growth of SMMC-7721 cells in a dose- and time-dependent manner. NK-lysin reduced the ability of cell migration, adhesion, and invasion. Based on gene and protein expression analysis, NK-lysin decreased *β*-catenin and MMP-2 expression. These results suggested that NK-lysin has anti-invasion and antimetastatic effects on hepatocellular carcinoma cells* in vitro* by reducing the level of the *β*-catenin and MMP-2.

## 1. Introduction

Hepatocellular carcinoma (HCC) is one of the most common malignant tumors, accounting for about 80% of the cases worldwide [1]. The main treatment methods of HCC include hepatectomy, chemotherapy, and liver transplantation, but the ability to invade and metastasize leads to recurrence of HCC [2]. However, in the current anticancer therapeutic approaches, there is lack of the drugs that specifically target cancer invasion and metastasis [3]. Therefore identification of agents that cause inhibition of local invasion and metastasis is new strategies for the treatment of HCC.

NK-lysin is effector protein peptides that is secreted by natural killer (NK) cells and cytotoxic T lymphocytes (CTLs). In 1995, Andersson first separated and purified NK-lysin from swine intestine [4]. Previous studies demonstrated that NK-lysin is an effective cationic antibacterial peptide that exhibits antibacterial properties and kills cancer cells without harming red cells [4, 5]. At present, our group has successfully developed a eukaryotic expression system of the recombinant NK-lysin* in vitro* and demonstrated that it can inhibit the proliferation of hepatocellular carcinoma cells [6]. However, it remains unclear whether the recombinant NK-lysin exerts anti-invasive and antimetastatic activities.

The purpose of the present study was to determine the potential molecular mechanisms of anti-invasion and antimetastatic effects of NK-lysin on HCC* in vitro*. Our results provide a basis for the future development of NK-lysin as an anticancer agent, which may contribute to developing new antineoplastic medicines.

## 2. Materials and Methods

### 2.1. Cell Line

The HCC cell line SMMC-7721 was purchased from the Cell Bank of Shanghai Chinese Academy of Sciences (Shanghai, China).

### 2.2. NK-lysin Preparation

The recombinant porcine NK-lysin was expressed using the* Pichia pastoris* system by our laboratory [6]. NK-lysin was dissolved in phosphate buffered saline (PBS). The solution was filtered through a 0.22-*μ*m filter and stored at -20°C.

### 2.3. Cell Proliferation Assay

SMMC-7721 cells in mid-log phase were seeded in a 96-well plate at a density of 4×10^4^ cells/well in 100 *μ*L of medium (Thermo Fisher Scientific, Waltham, USA) and incubated at 37°C and 5% CO_2_ for 24 hrs. The supernatant was discarded, and cells were treated with a series of NK-lysin concentrations (320, 160, 80, 40, 20, 10, 5, and 2.5 *μ*g/mL) for 24, 48, and 72 hrs using PBS as a control. The inhibition effect of NK-lysin on SMMC-7721 cells was analyzed using the MTT assay (Amresco, USA). After 24, 48, and 72 hrs, the supernatant was discarded, and 20 *μ*L of 5 mg/mL MTT was added to every well and incubated at 37°C for 4 hrs. The supernatant was discarded, and 150 *μ*L of DMSO (Solarbio, Beijing, China) was added to each well. The cells were incubated at 37°C for 20 min until the precipitate was completely dissolved. The spectrophotometric absorbance was measured using the microplate reader (ELx808; Gene Co., Ltd., Hong Kong, China) at 490 and 630 nm (absorbance 490 nm and reference 630 nm). Each independent experiment was performed in triplicate. The inhibition rate of cell proliferation (IR) was calculated as follows: IR = [1 - (OD490 nm- OD630 nm) treated/(OD490 nm - OD630 nm) nontreated control] × 100%.

### 2.4. Cell Wound-Healing Assay

The wound-healing assay was used to evaluate the cancer cell migration capability in 2D space. SMMC-7721 cells at mid-log phase were seeded in a 12-well plate at a density of 4×10^5^ cells/well in 1 mL of medium, and they were incubated at 37°C and 5% CO_2_ for 24 hrs. A scratch was made (along the width of the well) using 1-10 *μ*L sterile pipette tip. The supernatant was discarded and the cells were washed twice with PBS. The cells were then treated with various concentrations of NK-lysin (40, 20, and 10 *μ*g/mL) for 24 hrs and PBS was used as a control. The migrated distance was measured by Image J. Each independent experiment was performed in triplicate. The inhibition rate of cell migration (IR) was calculated as follows: IR = [(transport width of nontreated control−transport width of the experimental group migration)/transport width of nontreated control] × 100%.

### 2.5. Cell Adhesion Assay

A 96-well plate was coated with Matrigel (Corning Incorporated, New York, NY, USA) and incubated overnight. SMMC-7721 cells at mid-log phase were seeded in a 6-well plate at a density of 8×10^5^ cells/well in 2 mL of medium, and the cells were incubated at 37°C and 5% CO_2_ for 24 hrs. The supernatant was discarded, and the cells were treated with a series of NK-lysin concentrations (40, 20, and 10 *μ*g/mL) for 24 hrs and PBS was used as a control. After treatment, the cells were collected and seeded in a 96-well plate at a density of 2×10^4^ cells/well in 100 *μ*L medium and incubated at 37°C and 5% CO_2_ for 2 hrs. The supernatant was discarded and 20 *μ*L of 5 mg/mL MTT was then added to every well. The cells were incubated at 37°C for 4 hrs, supernatant was discarded, and 150 *μ*L DMSO was added to each well, incubated at 37°C for 20 min until the precipitate was completely dissolved. The spectrophotometric absorbance at 570 nm was measured using the microplate reader. Each independent experiment was performed in triplicate. The inhibition rate of cell adhesion (IR) was calculated as follows: IR = [(nontreated control OD value−experimental group OD value)/nontreated control OD value] × 100%.

### 2.6. Cell Transwell Assay

Invasion assays were used to investigate cancer cell migration in three-dimensional (3D) gels. The transwell upper chamber was coated with Matrigel and incubated at 37°C and 5% CO_2_ for 2 hrs. The SMMC-7721 cells were washed once with PBS and serum-free medium was added and then grown at 37°C and 5% CO_2_ for 12 hrs. Cells were collected and seeded in the transwell upper chamber (8.0 *μ*m/6.5 mm; Corning Incorporated, New York, NY, USA) at a density of 2×10^4^ cells/well in 200 *μ*L of medium containing various concentrations of NK-lysin (40, 20, and 10 *μ*g/mL) and PBS. The bottom chambers were filled with culture medium containing 20% fetal bovine serum (FBS). After incubation for 24 hrs, the cells on the upper surface of the well were removed, and the cells on the lower surface were fixed with cold methanol and stained with 0.4% crystal violet. For each experiment, the number of transmigrated cells in five random fields on the underside of the filter was counted and photographed. Each independent experiment was performed in triplicate. The inhibition rate of cell invasion (IR) was calculated as follows: IR = [(number of nontreated control invasive cell−number of the experimental group invasive cell)/number of nontreated control invasive cell] × 100%.

### 2.7. Real-Time Quantitative Polymerase Chain Reaction (qPCR) Assay

Total RNA was extracted using TRIzol reagent (Invitrogen, USA) according to the manufacturer's instructions. Purified RNA with an A260/A280 ratio of 1.8 to 2.0 was used. Total RNA (1*μ*g) was used for cDNA synthesis with random hexamer primers using a Prime Script RT reagent kit with gDNA Eraser (TaKaRa Bio, Co, Ltd, Dalian, China). qPCR was performed on 7500 Real Time PCR System (Life Technologies, USA) using the QuantiTect SYBR Green PCR kit (QIAGEN, Hilden, Germany) according to the manufacturer's instructions. The detected genes included MMP-2 (Accession number: NM_004530.5, F: TGATCTTGACCAGAATACCATCGA; R: GGCTTGCGAGGGAAGAAGTT) and *β*-catenin (Accession number: X87838, F: ACCTTTCCCATCATCGTGAG; R: GCTTTGGTTCACCAGTGGATT). The data were then normalized to the expression of *β*-actin (Accession number: NC_018913.2, F: AAATCGTGCGTGACATTAA; R: GGAAGGAAGGTTGGAAGAGAGC) mRNA. The PCR cycling conditions were as follows: 95°C for 5 min followed by 40 cycles of 95°C for 10 s, 60°C for 30 s, and 72°C for 6 s. Based on the melting curve analysis, the PCR product was demonstrated to be specific. The expression level of the target gene was compared with the control group, and the results were calculated using the 2-∆∆Ct method. Each RNA sample was measured in duplicate.

### 2.8. Western Blot Assay

The cells were lysed in 500 *μ*L of lysis buffer (APPLY GEN, Beijing, China) per well for 2 min on ice, and the lysates were collected in 1.5-mL centrifuge tubes. The lysates was added to 500 *μ*L of extraction buffer for 10 min on ice followed by centrifugation at 10000×g for 20 min at 4°C. The middle phases were harvested and 1 mL of ethanol was added and the sample was then centrifuged at 10000×g for 3 min at 4°C. The precipitate was collected and air-dried and 150 *μ*L of 2% sodium dodecyl sulfate (SDS) was added to dissolve the protein. The protein concentration was determined with a BCA Kit (BCA, Beyotime, Shanghai). Equal amounts of protein (50 *μ*g) were used for 8% sodium dodecyl sulfate-polyacrylamide gel electrophoresis (SDS-PAGE). Protein blots were transferred to polyvinylidene difluoride (PVDF) membranes (Millipore, Billerica, MA, USA). The membranes were blocked with 5% bovine serum albumin (BSA) in Tris-buffered saline with Tween-20 (TBST) for 2 hrs and incubated with primary antibodies (Cell Signaling Technology, Inc., USA) overnight at 4°C. The membranes were washed thrice with TBST (20% Tween-20) and incubated with the secondary antibodies (ComWin Biotech, Beijing, China) for 1 hr at 37°C and then washed again thrice with TBST. Immunoreactive bands were detected by enhanced chemiluminescence (ECL) and visualized by exposure to Kodak X-ray film (Eastman Kodak, Rochester, NY, USA). The protein expression levels were compared with the control based on the relative intensities of the bands.

### 2.9. Statistical Analysis

All data were analyzed by one-way analysis of variance (ANOVA) followed by Dunnett's multiple comparison test and P<0.05 was considered as statistically significant. The data were shown as means ± SEM and analyzed using GraphPad Prism5 software (GraphPad Software, Inc., San Diego, CA).

## 3. Results

### 3.1. NK-lysin Inhibits SMMC-7721 Cell Proliferation

**Figure 1:**
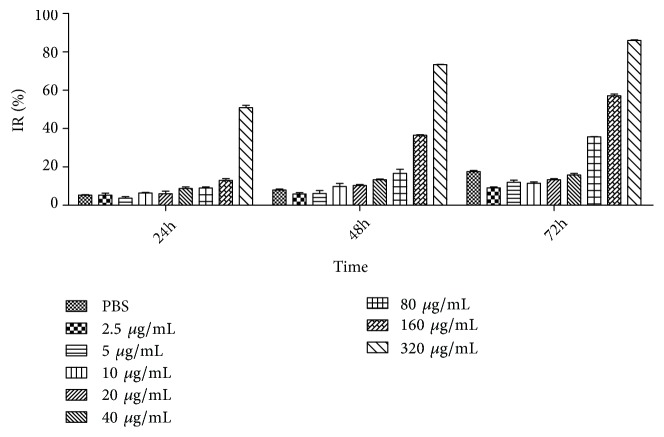
Proliferation inhibition effect of NK-lysin on SMMC-7721 cells (n=3).

We tested the proliferative inhibition effect of NK-lysin on the SMMC-7721 cell line using the MTT assay at different treatment intervals. The cellular proliferative inhibition rate of 320 ug/mL of NK-lysin was 49%, 73%, and 85% at 24, 48, and 72 hrs, respectively. This finding demonstrated that NK-lysin treatment significantly inhibited SMMC-7721 cell growth in both dose- and time-dependent manner at 24, 48, and 72 hrs (Figure 1).* In vitro *the percentages of cancer cell survival need to reach 85%-90% for detecting invasion and metastasis. The inhibition rate of the cells was less than 16% when SMMC-7721 cells were treated for 72 hrs with concentrations of NK-lysin less than 40 *μ*g/mL. This result suggested that this concentration and time point were suitable for detecting invasion and metastasis. Thus, we used doses of 40, 20, and 10 *μ*g/mL for the follow-up experiments.

### 3.2. Effect of NK-lysin on SMMC-7721 Cell Migration Ability

**Figure 2:**
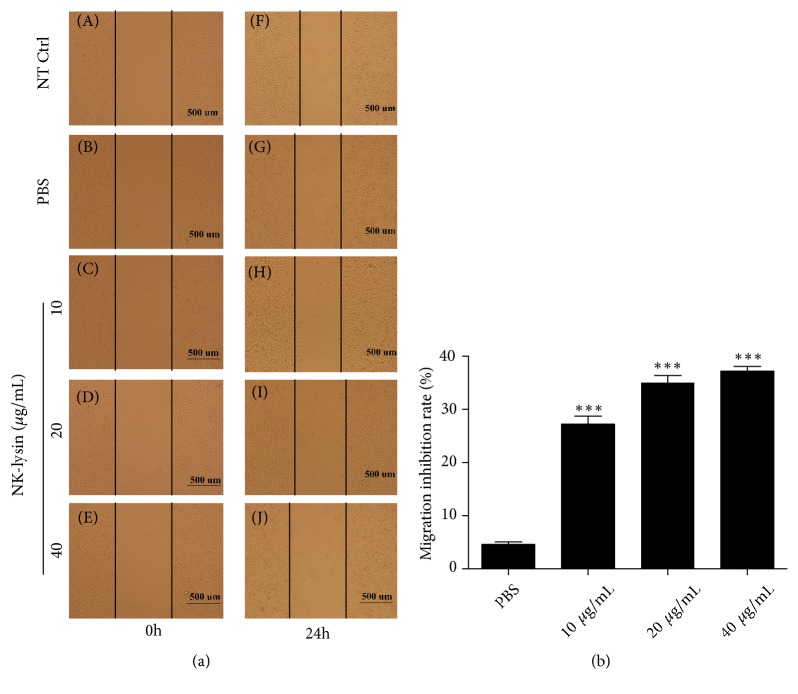
*The effect of NK-lysin on SMMC-7721 cell migration*. The scratch width of the different groups on SMMC-7721 cells after 24 hrs (a). Migration inhibition analysis of the various concentrations of NK-lysin and PBS (b). The migration inhibition rate of the NK-lysin group compared with the PBS group. The values are presented as the mean ± SEM (n=3), *∗∗∗P*<0.001 vs. the PBS group.

The migration ability of cancer cells is directly related with disease progression as higher migration rate results in reduced opportunities to heal cancer patients. We investigated the migration effects of NK-lysin on SMMC-7721 cells* in vitro*. The migration inhibition rates of NK-lysin-treated cells were 26.97±2.641%, 34.81±2.292%, and 36.96±1.511% at 10, 20, and 40 *μ*g/mL, respectively, whereas the migration inhibition rate of PBS-treated cells was 4.265±0.8175%. The migrated distance of NK-lysin-treated cells significantly decreased compared with the nontreated control (Figure 2(a)). The wound of nontreated control cells was almost closed, but the NK-lysin treated cells exhibited noticeable injury. Nevertheless, the migration inhibition rate did not change in the PBS-treated group compared to the nontreated control group (Figure 2(b)).

### 3.3. Effect of NK-lysin on the Adhesion Ability of SMMC-7721 Cells

**Figure 3:**
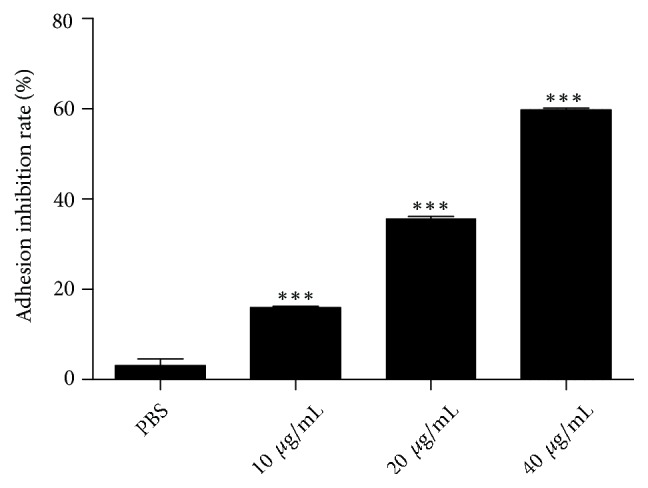
*The adhesion inhibition analysis of the different concentrations of NK-lysin and PBS*. Cells of various groups were incubated for 24 hrs. The adhesion inhibition rate of NK-lysin group significantly differed from the PBS group. The values are presented as the mean ± SEM (n=3), *∗∗∗P*<0.001 vs. the PBS group.

We investigated the effects of NK-lysin on SMMC-7721 cell adhesion* in vitro* because the adhesion ability of cancer cells has a direct positive correlation with invasion. The adhesion inhibition rates of NK-lysin-treated cells were 14.72±0.4838%, 34.73±0.9122%, and 59.58±0.4159% at 10, 20, and 40 *μ*g/mL, respectively, whereas the adhesion inhibition rate of PBS-treated cells was 3.143±3.201%. The cell adhesion ability was significantly decreased in the NK-lysin-treated cells compared with nontreated control cells. However, the adhesion inhibition rate did not change in the PBS-treated group compared to the nontreated control group (Figure 3).

### 3.4. Invasion Ability Effect of NK-lysin on SMMC-7721 Cells

**Figure 4:**
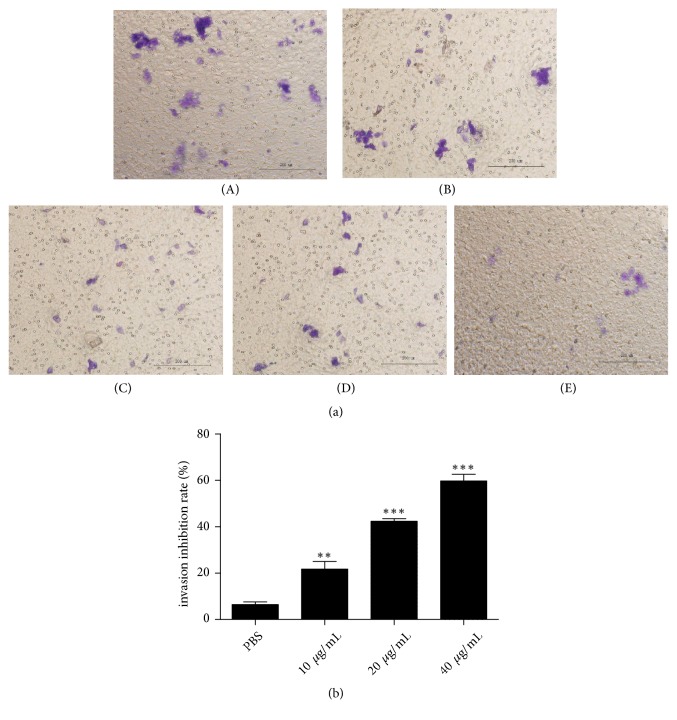
*The invasion effect of NK-lysin on SMMC-7721 cells*. Cells on the lower surface were fixed with cold methanol and stained with 0.4% crystal violet (a). (A): Nontreated control group, (B): PBS group, (C): 10 *μ*g/mL NK-lysin group, (D): 20 *μ*g/mL NK-lysin group, and (E): 40 *μ*g/mL NK-lysin group. The analysis of invasion inhibition rate of the different NK-lysin concentrations (b). The invasion inhibition rate of the NK-lysin group significantly differed from the PBS group. The values are presented as the mean ± SEM (n=3), *∗∗P*<0.01, *∗∗∗P*<0.001 vs. the PBS group.

Invasion is a canonical feature of cancer cells, and it is a prerequisite for cancer metastasis. Therefore, we investigated the effects of NK-lysin on the invasion of SMMC-7721 cells* in vitro* (Figure 4(a)). The invasion inhibition rates of NK-lysin-treated cells were 19.71±4.267%, 41.31±0.6577%, and 59.87±3.159% at 10, 20, and 40 *μ*g/mL concentration of NK-lysin, respectively, while the invasion inhibition rate of PBS-treated cells was 8.650±1.526% compared with the nontreated control cells. The invasion ability was significantly decreased in the NK-lysin-treated groups compared with the nontreated control SMMC-7721 cells. However, the invasion inhibition rate ability did not changed in the PBS-treated group compared to the nontreated control group (Figure 4(b)).

### 3.5. Effect of NK-lysin on *β*-Catenin and MMP-2 Expression in SMMC-7721 Cells

**Figure 5:**
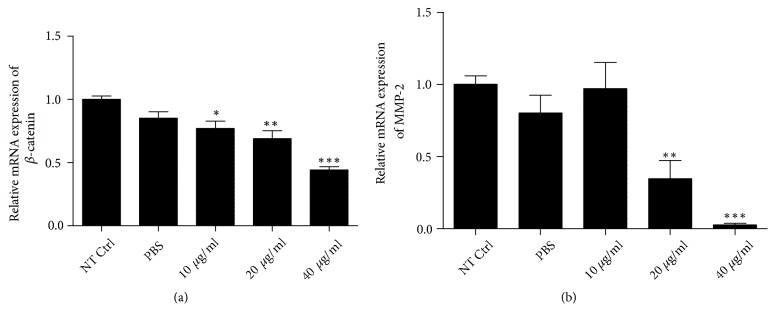
*The effects of NK-lysin on relative mRNA expression levels in SMMC-7721 cells*. Cells of different groups were incubated for 72 hrs, and qRT-PCR was used to evaluate mRNA expression levels. Relative mRNA expression of *β*-catenin (a) and MMP-2 (b). The values are presented as the mean ± SEM (n=3), *∗P*<0.05, *∗∗P*<0.01, and *∗∗∗P*<0.001 vs. the NT Ctrl group.

**Figure 6:**
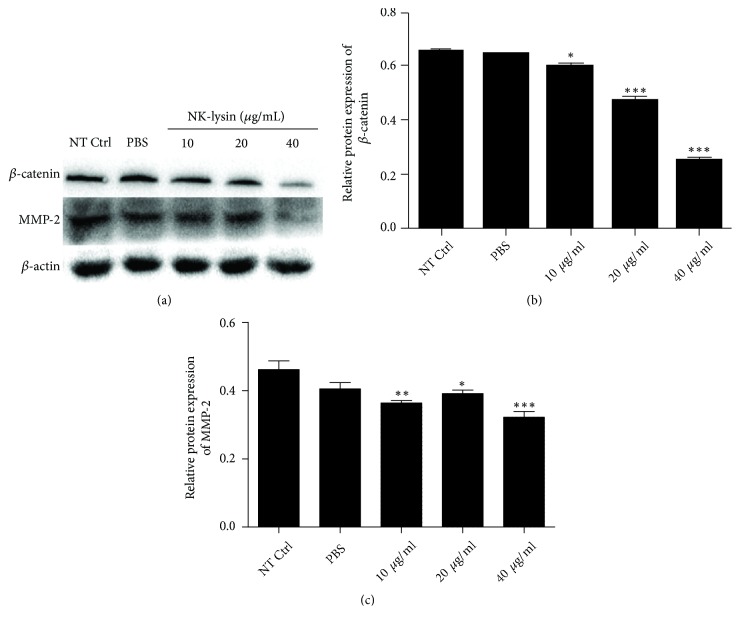
*The effects of NK-lysin on protein expression levels in SMMC-7721 cells*. Cells of different groups were incubated for 72 hrs, and Western blot analysis detected the levels of protein expression. *β*-actin was used as an internal control (a). Relative protein expression of *β*-catenin (b) and relative protein expression of MMP-2 (c). The values are presented as the mean ± SEM (n=3), *∗*P<0.05, *∗∗*P<0.01, and *∗∗∗*P<0.001 vs. NT Ctrl group.

The extracellular matrix (ECM) is degraded by matrix metalloproteinases (MMPs), which is an important step in cancer invasion and metastasis. MMP-2 is a key member of the MMP family that can degrade the ECM. The present results showed that *β*-catenin and MMP-2 were highly expressed in cancer cells [7, 8]. Thus, we investigated alterations of *β*-catenin and MMP-2 expression in NK-lysin treated cells. *β*-catenin and MMP-2 mRNA expression levels were significantly decreased compared with nontreated control SMMC-7721 cells. However, the mRNA expression levels of *β*-catenin and MMP-2 did not change in the PBS-treated group compared to the nontreated control group (Figure 5). Western blot results were consistent with the mRNA expression levels (Figure 6).

## 4. Discussion

Our previous study has found that NK-lysin suppressed the proliferation and reduced the filopodium formation of hepatocellular carcinoma [6]. The filopodium was considered to be related with the ability of cancer cells to metastasize [9]. Therefore, we examined the effects of NK-lysin on anti-invasion and antimetastasis with SMMC-7721 hepatocellular carcinoma. Firstly, the effects of NK-lysin on SMMC-7721 cells proliferation were tested by the MTT assay. The results indicated that NK-lysin inhibit the proliferation of SMMC-7721 cells in concentration and time-dependent manner, and NK-lysin significantly decreased the migration of cells in wound-healing assay, the invasion ability of the SMMC-7721 cells in invasion assay, and adhesion ability of the SMMC-7721 cells in cell adhesion assay. It indicates that NK-lysin can significantly inhibit the invasion and metastasis of HCC cells.

Aberrant activation of the Wnt/*β*-catenin signalling pathway has a close relationship with cancer initiation, progression, and metastasis [7, 10, 11]. Invasion and metastasis of HCC have been inhibited by decreasing Wnt/*β*-catenin signaling [12, 13]. MMPs, a very important proteolytic enzyme, are involved in the cancer cells invasion and metastasis [8, 14]. MMPs efficiently degrade extracellular matrix components in tissue surrounding a tumor, which results in the entry and survival of cancer cells in the circulation, lymphatic, or peritoneal spaces, and arrest in a distant target organ [2, 15]. *β*-catenin and MMP-2 play critical roles in cancer cell invasion and metastasis and these proteins are overexpressed in cancer. In this study, we demonstrated NK-lysin could significantly reduce *β*-catenin and MMP-2 expression of SMMC-7221 cells at the mRNA and protein levels. The results indicated that NK-lysin may inhibit HCC invasion and metastasis by suppressing the expression of *β*-catenin and MMP-2.

## 5. Conclusions

The present study indicated that NK-lysin has significant anti-invasion and antimetastasis effects on HCC cells. Further in-depth research will be performed because NK-lysin is a potential candidate compound for HCC treatment to help develop new antimicrobial peptides as anticancer drugs.

## Data Availability

The data used to support the findings of this study are available from the corresponding author upon request.
